# Evaluation of the reliability of the criteria for assessing prescription quality in Chinese hospitals among pharmacists in China

**DOI:** 10.1186/s12913-022-07854-1

**Published:** 2022-04-07

**Authors:** Xiaofang Tan, Jun Zhang, Dongshi Gu, San Ran, Tongtong Gu, Xiaowen Lin, Enxiang Tao, Tetsuya Asakawa, Huan Fang

**Affiliations:** 1 Department of Pharmacy, Jinshan Hospital of Fudan University, No. 1508 Longhang Road, Shanghai, 201508 PR China; 2 Department of Pharmacy, Jinshan Central Hospital, Shanghai, 201500 China; 3 Department of Neurology, The Eighth Affiliated Hospital, Sun Yat-Sen University, Shennanzhong Road 3025, Shenzhen, 518033 Guangdong Province China; 4 Research Base of Traditional Chinese Medicine Syndrome, Fujian University of Traditional Chinese Medicine, Fuzhou, 350122 China

**Keywords:** Reliability, Criteria for assessing prescription quality in Chinese hospitals (CAPQCH), Prescription comments, Cohen’s kappa, Fleiss’ kappa

## Abstract

**Background:**

The reliability of Criteria for Assessing Prescription Quality in Chinese Hospitals (CAPQCH) has never been rigorously verified. This study was designed to verify the reliability of the CAPQCH among pharmacists in China.

**Methods:**

Fourteen pharmacists, 5 from hospitals and 9 from the communities were recruited. We randomly selected 200 prescriptions, and made the testing prescriptions including appropriate and inappropriate testing prescriptions. Pharmacists assessed these testing prescriptions according to criteria in CAPQCH. Three test sets (Set 1, Set 2, and Set 3) were evaluated at 6-month intervals. Before administration of Set 3, pharmacists were informed that achievement on Set 3 would be reflected in their performance appraisal. We also evaluated the performance based on prescription comments before and after combining several confusing criteria. Cohen’s Kappa statistic, Fleiss’ Kappa statistic, and accuracy were employed to evaluate reliability among pharmacists.

**Results:**

Median values of Cohen’s Kappa were 0.61 in Set 1, 0.66 in Set 2, and 0.80 in Set 3; reliability is thus substantial. Our data indicate no significant differences between Set 1 and Set 2, whereas Set 3 indicates significantly improved performance. Moreover, combinations of confusing criteria contributed little to improvement of performance in prescription comments.

**Conclusion:**

Our results verified the reliability of CAPQCH application by working pharmacists. Adding performance based on prescription comments to personal appraisals was effective in improving the quality of prescription comments. These findings may be useful when future modification of the CAPQCH is considered. Moreover, this study contributes to improving the understanding of the prescription assessment situation in China.

**Supplementary Information:**

The online version contains supplementary material available at 10.1186/s12913-022-07854-1.

## Background

Inappropriate prescription is common in aging populations and is a global public health concern because it leads to negative clinical outcomes, such as increasing adverse drug events, morbidity, and costs of hospitalization and care. The global population is aging, and reduction of numbers of inappropriate prescriptions to improve safe drug use is a challenge that must be faced by governments worldwide. Our earlier studies indicated that improving the spontaneous reporting system [1] may contribute to reducing medication errors [2], and thereby improving prescription quality. Establishing compelling screening tools based on prescription comments and issued by public health authorities might play a crucial role in reducing inappropriate prescription. The most important tool for identifying inappropriate prescriptions is the Beers criteria, first reported in 1991 [3], and updated in 2003 [4]. Later, several screening tools were developed and used, such as the Screening Tool of Older Peoples Prescriptions (STOPP) and the Screening Tool to Alert doctors to Right Treatment (START) [5], the Pharmaceutical Care Network Europe (PCNE) [6], the Medication Appropriateness Index (MAI) [7], and the National Coordinating Council for Medication Error Reporting and Prevention (NCC MERP) [8]. The Chinese government also issued the Criteria for Assessing Prescription Quality in Chinese Hospitals (CAPQCH) in 2010 [9]. This guidance is a 28-item criterion that guides development of prescription comments that must be reported by pharmacists (Table S1).

Reliability of prescription comments is a critical issue. The reliability of the widely used tools, such as Beer’s criteria, STOPP, START, PCNE, and MAI, has been verified. Inter-rater reliability is typically used to assess reliability of such tools. Higher inter-rater reliability indicates higher consistency among different examiners. This approach can provide compelling evidence for overall reliability of screening tools. Ryan et al. evaluated inter-rater reliability of STOPP and START with 10 pharmacists from hospitals and the communities. Finally, inter-rater reliability of STOPP and START was judged to be “good” for both pharmacists in hospitals and the communities [10]. Hohmann et al. assessed inter-rater reliability of PCEN with nine pharmacists and three pharmacy interns. They found that inter-rater reliability was substantial for main criteria and moderate for subcriteria [11]. Stuijt et al. evaluated inter-rater reliability of MAI with eight raters and found an overall agreement rate of 83%, the overall chance-adjusted inter-rater agreement was moderate (Kappa = 0.47), and the overall intra-group agreement was excellent (Kappa over 0.84). The authors concluded that MAI is a reliable tool for evaluation of prescription [7]. Inter-rater reliability is thus a powerful tool for evaluating the overall reliability of a screening tool.

The reliability of CAPQCH has never been rigorously verified. During practical application, some reports in Chinese documented that some criteria, namely “unsuitable usage and dosage,” “no or incomplete clinical diagnosis,” “improper drug combination,” and “incompatibility or bad interactions between drugs,” might be confusing [12, 13]. We designed our study based on this knowledge to 1) verify inter-rater reliability of CAPQCH, 2) assess the relationship of performance of CAPQCH and personal performance appraisals, and 3) examine the impact of a combination of reported confusing criteria on consistency. Our study provides evidence for the reliability of CAPQCH and will be beneficial to the future modification of this tool. The findings of this study will contribute to improving the understanding of the prescription assessment situation in China as well as the methodology for evaluating the reliability using the inter-rater study.

## Methods

### Participants

Fourteen pharmacists were recruited, 5 from the hospital sector (recorded as “HPs” in this study) and 9 from the community sector (recorded as “CPs” in this study). Subjects work in hospitals within the Jinshan district in the south of Shanghai city, China (Table 1). The working contents between HPs and CPs are different in this city. The HPs are mainly in charge of the clinical pharmacy in a local hospital. Prescription assessment is their routine work. Whereas the CPs are working in the community (community medical service). They mainly provide pharmaceutical service to a specific community region and perform prescription assessment for the doctors in community. Because the diseases and patients were different between hospital and community, the prescriptions they reviewed are slightly different. Because the use of the Criteria for Assessing Prescription Quality in Chinese Hospitals is part of a standard practice in the hospital, this study has been granted an exemption from requiring ethics approval by the Ethics Committee of Jinshan Hospital of Fudan University. The informed consent is also waived by the Ethics Committee of Jinshan Hospital of Fudan University. All the participants were informed the purposes of the study prior to data collection.

**Table 1:** Characteristics of raters

Number of raters	Position	Gender	Age	Experience in pharmacy (years)
1	Hospital	Female	40	19
2	Hospital	Female	34	14
3	Hospital	Female	34	10
4	Hospital	Male	34	10
5	Hospital	Female	28	5
6	Community	Female	33	11
7	Community	Male	47	29
8	Community	Female	50	31
9	Community	Female	41	20
10	Community	Female	35	16
11	Community	Female	38	16
12	Community	Female	33	11
13	Community	Female	53	30
14	Community	Male	34	13

### Testing prescriptions

Prescriptions for the study were first selected randomly by two experienced pharmacists from the electronic prescription system. A total of 200 prescriptions written from September to October 2019 were selected, which were then evaluated and modified to form two serials of testing prescriptions, namely “appropriate” prescriptions and “inappropriate” prescriptions using CAPQCH. All inappropriate prescriptions were modified to ensure that there is only “one mistake” in it. Mistakes were chosen to match expected professional knowledge and experience of raters. The “mistake” was defined according to CAPQCH and a previous study concerning CAPQCH [14] as 1) irregular prescription prescriptions, such as no or incomplete clinical diagnosis, no physicians’ signature on the prescription, or inconsistent with sample signature, nonstandard prescription paper, no signature and date for prescription revision, no reason and new signature for overdose use, and outpatient prescriptions for more than 7 days or emergency prescriptions more than 3 days without special conditions, and 2) inappropriate medication, such as unsuitable usage and dosage, prescriptions without indication, improper drug selection, or drug interactions, improper drug combination, incompatibility or dangerous interactions, and improper route of administration. Standard answers for test prescriptions were developed by the two pharmacists and finally checked and reviewed by an expert group of three senior pharmacists. Consensus was reached for all testing prescriptions and standard answers among pharmacists and experts before the testing began. We defined standard answers as “rater A”.

### Setting

All recruited pharmacists participated in three tests, given in September 2019 (Set 1), March 2020 (Set 2), and September 2020 (Set 3). The pharmacists assessed test prescriptions and identified “mistakes” according to guidance of prescription comments. Answers (raters 1–14) were then reviewed by the expert group. Set 1 and Set 2 tests were free from any connection to pharmacists’ appraisal. Before Set 3 (1 month before implementation), all pharmacists were informed that their achievement with Set 3 would be recorded in the assessment of the quality of their prescription comments, which is related to their performance appraisal. A pharmacist who achieved good performance on the test would earn a “good” score on his/her appraisal. Conversely, poor performance would earn a “poor” score. The performance appraisal is regularly associated with the ranking of their department in the local public health administrative department.

### Statistics

All the answers were entered into a database and analyzed using SPSS software (V26.0.0, IBM, IL, USA). Cohen’s Kappa statistic was used to assess the consistency between raters 1–14 and rater A [15]. The degree of reliability was defined as 1) poor, Kappa ≤0.20; 2) fair, 0.21 ≤ Kappa ≤0.40; 3) moderate, 0.41 ≤ Kappa ≤0.60; 4) substantial, 0.61 ≤ Kappa ≤0.80; and 5) excellent, 0.81 ≤ Kappa ≤1.00 [10]. The proportion of positive agreement (Ppos) and the proportion of negative agreement (Pneg) were also evaluated [16].

Overall consistency within a single test was assessed using Fleiss’ Kappa statistic. Accuracy was calculated, by pharmacists, as the number of “right raters” (raters in agreement with rater A) divided by the number of ratings.

According to the previous studies [8, 12], some criteria might cause confusion during testing. We thus combined these terms. For example, criteria “inappropriate selection of indications” and “no or incomplete clinical diagnosis” and criteria “improper drug combination” and “incompatibility or bad interactions between drugs” were combined. We assessed changes in reliability with and without these combinations.

Normality of data distributions was assessed using the Kolmogorov–Smirnov test. Normally distributed data with Kolmogorov–Smirnov values over 0.05 were submitted for Levene’s test to verify homogeneity of variances. Paired t-tests were then used to compare differences between raters and rater A for each pharmacist. Independent sample t-tests were used to compare the difference between HPs and CPs. Data not normally distributed were compared using the Wilcoxon test. *p* < 0.05 was considered statistically significant.

## Results

Seventy-nine percent of raters were female, five pharmacists were from hospitals, and nine were from the community. The average age was 38.14 ± 1.94 y (first quartile–third quartile: 33.75–42.50 y). Average work experience was 16.79 ± 2.18 y (first quartile–third quartile:10.75–22.25 y) (Table 1).

Inter-rater reliability of pharmacists was evaluated using Cohen’s Kappa statistic. Median values of the statistic for sets 1, 2, and 3 were 0.61 (95% CI, 0.54–0.67), 0.66 (0.55–0.75), and 0.80 (0.74–0.87), respectively. These values exceed 0.6, indicating good reliability for all tests. An increasing tendency in the statistic is seen from sets 1 to 3. No significant difference was found between Set 1 and Set 2. The statistic for Set 3 was significantly greater than those for Set 1 (*p* < 0.001) and Set 2 (*p* < 0.05). Further, the lowest values of Cohen’s Kappa statistic were 0.33 (95% CI, 0.15–0.48) in Set 1 and 0.42 (0.25–0.58) in Set 2, indicating a “moderate” Cohen’s Kappa statistic in the first two tested sets. Accordingly, proportions of values lower than 0.6 were 50% in Set 1, 29% in Set 2, and 0% in Set 3 (Table S2). No significant differences between HPs and CPs were seen, although values of hospital pharmacists were greater than those of pharmacists from the community. The largest Kappa statistic was found for HPs in the Set 3 test—0.86 (0.75–0.88)—achieving “excellent”. The lowest was for CPs in the Set 1 test—only 0.59 (0.48–0.65)— “moderate”. When Kappa statistics among sets were compared, Set 3 was significantly elevated over sets 1 and 2 in CPs (*p* < 0.05), but was significantly higher than only Set 1 (*p* < 0.05) in HPs (Table 2).

**Table 2:** Comparison of reliability between pharmacists in hospitals and those in the community on three sets of prescriptions using Cohen’s Kappa

Comparators	Ppos	Pneg	Median Kappa (95% Cl)
**Set 1**			
SA			
HPs	0.64	0.98	0.63 (0.59–0.74)
CPs	0.61	0.98	0.59 (0.48–0.65)
**Set 2**			
SA			
HPs	0.76	0.99	0.75 (0.62–0.81)
CPs	0.66	0.99	0.64 (0.46–0.71)
**Set 3**			
SA			
HPs	0.86	0.99	0.86 (0.75–0.88) **∆**
CPs	0.76	0.99	0.75 (0.73–0.86) **∆, #**

*Ppos* Proportion of positive agreement, *Pneg* Proportion of negative agreement, *HPs* Hospital pharmacists, *CPs* Community pharmacists, *SA* Standard answers

∆ means *p* < 0.05, Set 3 vs. Set 1; # means *p* < 0.05, Set 3 vs. Set 2

Evaluation of overall reliability using Fleiss’ Kappa had the same results as those of reliability indicated by Cohen’s Kappa. Values of Fleiss’ Kappa were lowest in the Set 1 test and increased for Set 2 and increased further for Set 3 for both CPs and HPs.

No significant difference was found between HPs and CPs for accuracy among Set results. Overall, no significant difference was found between sets 1 and 2; however, data from Set 3 were significantly greater than either Set 1 or Set 2 (*p* < 0.05) (Table 3).

**Table 3:** The inter-rater agreement among 14 pharmacists for three sets of prescription evaluations

Comparators	TPs	HPs	CPs
**Inter agreement Fleiss’ Kappa (95% Cl)**			
** Set 1**	0.44 (0.40–0.48)	0.62 (0.50–0.73)	0.38 (0.32–0.44)
** Set 2**	0.51 (0.47–0.54)	0.62 (0.50–0.73)	0.47 (0.41–0.53)
** Set 3**	0.68 (0.64–0.72)	0.69 (0.57–0.80)	0.69 (0.63–0.75)
**Accuracy Rate (%)**			
** Set 1**	61.22	67.14	57.94
** Set 2**	65.76	73.10	61.69
** Set 3**	80.54 **∆, #**	82.76 **∆**	79.31 **∆, #**

*TPs* Total pharmacists, *HPs* Hospital pharmacists, *CPs* Community pharmacists

∆ means *p* < 0.05, Set 3 vs. Set 1; # means *p* < 0.05, Set 3 vs. Set 2

Thus, the inclusion of performance on prescription comments in personal performance appraisals may enhance the quality of comments.

Reliability evaluated using the Cohen’s Kappa statistic and accuracy after combining confusing criteria based on a previous study [8] indicated no impact in the Set 3 test and small increases in Set 1 and Set 2 tests for TPs. This tendency was unchanged regardless of which test criteria were combined: Set 3 > Set 2 > Set 1 (Table 4). Tendencies in accuracy were in complete agreement with Cohen’s Kappa (Table 5). These data suggested that when performance of consistency is poor (Set 1 and Set 2), combination of the confusing criteria might be able to mildly improve reliability. When the performance of consistency is satisfactory (Set 3), the improvement that results from criterion-adjustment is quite limited.

**Table 4:** The inter-rater reliability among 14 pharmacists for three sets of prescriptions compared using Cohen’s Kappa statistics after combining confusing criteria

	TPs	HPs	CPs
**Median Kappa 95% Cl)**			
**Set1**			
Before combination	0.61 (0.54–0.67)	0.63 (0.59–0.74)	0.59 (0.48–0.65)
After combination	0.63 (0.57–0.70)*	0.63 (0.61–0.76)	0.59 (0.50–0.67)
**Set2**			
Before combination	0.66 (0.55–0.75)	0.75 (0.62–0.81)	0.64 (0.46–0.71)
After combination	0.68 (0.57–0.75)*	0.75 (0.64–0.84)	0.64 (0.52–0.71)
**Set3**			
Before combination	0.80 (0.74–0.87) ∆, #	0.86 (0.75–0.88) ∆	0.75 (0.73–0.86) ∆, #
After combination	0.80 (0.74–0.89) ∆, #	0.86 (0.77–0.89) ∆	0.75 (0.73–0.86) ∆, #

*TPs* Total pharmacists, *HPs* Hospital pharmacists, *CPs* Community pharmacists, *CI* Confidence interval. * means *p* < 0.05, before vs. after combination; ∆ means *p* < 0.05, Set 3 vs. Set 1; # means *p* < 0.05, Set 3 vs. Set 2

**Table 5:** Accuracy among 14 pharmacists for three sets of prescriptions after combining confusing items

	TPs	HPs	CPs
**Accuracy Rate (%)**			
**Set1**			
Before combination	61.22	67.14	57.94
After combination	62.76*	68.57	59.52
**Set2**			
Before combination	65.76	73.10	61.69
After combination	67.24*	75.17	62.84
**Set3**			
Before combination	80.54∆, #	82.76∆	79.31∆, #
After combination	81.03∆, #	84.14∆	79.31∆, #

*TPs* Total pharmacists, *HPs* Hospital pharmacists, *CPs* Community pharmacists, *CI* Confidence interval. * means *p* < 0.05, before vs. after combination; ∆ means *p* < 0.05, Set 3 vs. Set 1; # means *p* < 0.05, Set 3 vs. Set 2

## Discussion

We verified inter-rater reliability of CAPQCH with 14 pharmacists from Jinshan district using three sets of prescriptions. Cohen’s Kappa and Fleiss’ Kappa statistics were used to evaluate ratings. Cohen’s Kappa statistic along with accuracy can be used to evaluate the quality of prescription comments and to compare performance between CPs and HPs. The Fleiss’ Kappa statistic can be used to evaluate overall performance on prescription comments. This statistic was used to evaluate overall consistency among pharmacists and to explore dynamic changes in consistency among raters. Median values of Cohen’s Kappa were 0.61 for Set 1, 0.66 for Set 2, and 0.80 for Set 3. Inter-rater reliability was substantial. No significant differences in values of Kappa statistics were observed between Set 1 and Set 2; however, the statistic for Set 3 was significantly higher than statistics for the other two sets. Interestingly, evaluation of overall reliability indicated that the Fleiss’ Kappa statistic in Set 3 was improved for both CPs and HPs. Thus, associating performance using CAPQCH with performance appraisals will significantly increase reliability. In addition, the combination of reported “confusing” criteria contributes little to improving reliability. To the best of our knowledge, this study is the first to verify the reliability of CAPQCH and provides compelling evidence for its application, as well as any future modification.

A traditional inter-rater test was performed to evaluate consistency among 14 pharmacists by comparing their responses (raters 1–14) to standard answers (rater A) (Tables 2 and S2). Despite possible differences in work experience, the professional abilities of CPs and HPs did not differ for either Kappa statistics or accuracy. After connecting performance with CAPQCH to their personal appraisals (Set 3), the Kappa statistics for CPs and HPs significantly increased. The quality of prescription comments can be improved if performance was associated with appraisals. All pharmacists paid more attention to prescription comments and might take measures to increase their knowledge to achieve better performance. Perhaps these possibilities explain performance on Set 3. In comparison with previous studies and despite different experimental approaches, Kappa statistics in the present study (Set 1 and Set 2, non-intervention) are consistent with NCC MERP, approximately 0.61–0.63 [8], but lower than values from STOPP and START, typically over 0.85 [10]. This difference might be explained by the objectivity and specificity of STOPP and START, which reduce the subjectivity of the pharmacists during the preparation of prescription comments, thus achieving better consistency [17, 18].

Evaluation of overall consistency using the Fleiss’ Kappa statistic produced analogous results (Table 3). No significant difference was found between CPs and HPs for Fleiss’ Kappa statistic or accuracy. Accuracy for Set 3 was significantly higher than those for sets 1 or 2. The Fleiss’ Kappa statistic exhibited the same tendency, but differences did not reach significance. Again, relating quality of prescription comments and personal performance appraisals will remarkably improve the quality of comments.

With respects to the influence of “confusing items”. Finally, our results imply that these criteria have a little effect on performance for prescription comments. In sets 1 and 2, only TPs displayed significant changes (Tables 4 and 5). We believe that the significant increase in Kappa statistics and accuracy in Set 3 is due to connecting prescription comments with appraisals, rather than combining confusing criteria. This conclusion implies that reinforcing education and training might improve the abilities of pharmacists to implement CAPQCH. These issues require further investigation in future studies. Conversely, our data indicate limited influence of “confusing criteria” on the reliability of the current version of CAPQCH.

In the light of the findings of the present study, we understand that CAPQCH is a reliable tool to evaluate the potentially inappropriate prescription in China. It can be widely used in both HPs and CPs. Adding quality of prescription comments to personal performance appraisals of the pharmacists might improve the performance for the prescription comments, which should be noticed by those who are in charge of the hospital management. In addition, the “confusing items” seldom affected the performance for prescription comments, which should be taken into account in the future modification of the CAPQCH.

There are several limitations in this study. First, regarding the recruitment of pharmacists, although we had recruited all the pharmacists working in our hospital (five) and our community (nine), the sample size was not determined by a strict statistical method. This might lead a sampling bias. In addition, the small size of the participants might lead a biased conclusion. Second, we compared the potentially inappropriate medications between CAPQH and the other tools like NCC MERP, STOPP, and START directly using Kappa values. CAPQH is a list of items to check for formal content. Simply comparison of the Kappa values between CAPQH and the other tools might lead a biased conclusion because the purpose and contents of each tool are very different. We need to consider the results of CAPQH in light of its content. These issues will be fully considered and improved in our future investigations.

## Conclusions

This study verified the reliability of CAPQCH using a traditional inter-rater reliability test, which indicated that CAPQCH is a reliable tool to classify the inappropriate prescription in China. Our results suggest a little influence of reported confusing criteria. These items should be considered in the future modification of the CAPQCH. Instead, adding quality of prescription comments to personal performance appraisals might be more useful for improving comments. These findings contribute to our understanding of reliability of CAPQCH and may be useful for future modifications. In addition, this study contributes to improving the understanding of the prescription assessment situation in China.

## Supplementary Information

**Additional file 1: Table S1.** CAPQCH items. **Table S2.** The inter-rater reliability of criteria between rater A and raters 1–14 for three sets of prescriptions using Cohen’s Kappa statistics.

## Data Availability

The datasets used and/or analyzed during the current study are available from the corresponding author on reasonable request.

## Abbreviations

CAPQCH: Criteria for Assessing Prescription Quality in Chinese Hospitals
CPs: Pharmacists from the community sector
HPs: Pharmacists from the hospital sector
MAI: Medication Appropriateness Index
NCC MERP: National Coordinating Council for Medication Error Reporting and Prevention
PCNE: Pharmaceutical Care Network Europe
START: Screening Tool to Alert doctors to Right Treatment
STOPP: Screening Tool of Older Peoples Prescriptions
