# Supplementary material for: Evaluation of the reliability of the criteria for assessing prescription quality in Chinese hospitals among pharmacists in China
Source: BMC Health Serv Res. 2022 Apr 7;22:455. doi: 10.1186/s12913-022-07854-1 (PMC8988408; doi:10.1186/s12913-022-07854-1)
Supplement: Supplementary file 1 — Additional file 1: Table S1. CAPQCH items. Table S2. The inter-rater reliability of criteria between rater A and raters 1–14 for three sets of prescriptions using Cohen’s Kappa statistics. [file 12913_2022_7854_MOESM1_ESM.docx]

**Supplementary materials**

**Table S1. CAPQCH items**

| **Part I Irregular prescription** |
| --- |
| 1-1. Missing, improper, or illegible writing of the previous record, text, and postscript. |
| 1-2. Irregular doctor’s signature or seal or inconsistency between signature or seal and archived samples. |
| 1-3. Suitability of prescription unaudited by a pharmacist (lack of pharmacist’s signature at the review, allocation, check, and dispensing columns in prescription postscripts or no two signatures when a single pharmacist is on duty). |
| 1-4. Without writing age in days or age in months in the prescription for a newborn or infant. |
| 1-5. Mixed western medicine, patented Chinese medicine, and Chinese herbal medicine on one prescription. |
| 1-6. Prescription using an abnormal drug name. |
| 1-7. Dose, drug specification, quantity, and unit are nonstandard or unclearly written. |
| 1-8. Using vague words such as “as prescribed” and “use at one's convenience” for usage and dosage. |
| 1-9. No signature and modified date in a modified prescription or no written reasons for overdose or two signatures. |
| 1-10. Diagnosis without clinical diagnosis or incomplete diagnosis. |
| 1-11. Over five medicines in one outpatient prescription. |
| 1-12. The dose of medicine over 7 days in an outpatient prescription or over 3 days in an emergency prescription without special reasons or an extension of medicine for chronic diseases without stating reasons. |
| 1-13. Prescribing specially managed drugs, such as narcotics, psychotropics, medical toxicants, and radiopharmaceuticals without strictly complying with rules. |
| 1-14. Prescription of antibiotics without strictly complying with rules. |
| 1-15. Prescription of the Chinese herbal medicines without complying with the order of “principal, subordinate, adjuvant, and guide” or without a clear description of dispensing and decoction. |
| **Part II Inappropriate prescription** |
| 2-1. The indications are inappropriate |
| 2-2. The selection of drugs is not appropriate |
| 2-3. The dosage form or route of administration is not appropriate |
| 2-4. National essential medicines are not preferred without good reason |
| 2-5. The usage and dosage of drugs is not appropriate |
| 2-6. The combination of medicine is not appropriate |
| 2-7. The situation of repeated medication |
| 2-8. Drug use has incompatibility or adverse interaction |
| 2-9. Other inappropriate situations of drug use |
| **Part III Hypernormal prescription** |
| 3-1. Prescription without indications. |
| 3-2. Unreasonable prescription of costly drugs.  3-3. Prescription violates the instruction. |
| 3-4. Unreasonable prescription of the drugs with the same pharmacological effects. |

**Table S2. The inter-rater reliability of criteria between rater A and raters 1**–**14 for three sets of prescriptions using Cohen’s Kappa statistics.**

| **Rater combination** | **A** | **B** | **C** | **D** | **Ppos** | **Pneg** | **Kappa (95% Cl)** |
| --- | --- | --- | --- | --- | --- | --- | --- |
| **Set 1** |  |  |  |  |  |  |  |
| Rater A*rater 1 | 19 | 9 | 9 | 635 | 0.68 | 0.99 | 0.67(0.5-0.80) |
| Rater A*rater 2 | 18 | 10 | 10 | 634 | 0.64 | 0.98 | 0.63(0.46-0.76) |
| Rater A*rater 3 | 23 | 5 | 5 | 639 | 0.82 | 0.99 | 0.81(0.68-0.92) |
| Rater A*rater 4 | 18 | 10 | 10 | 634 | 0.64 | 0.98 | 0.63(0.46-0.76) |
| Rater A*rater 5 | 16 | 12 | 12 | 632 | 0.57 | 0.98 | 0.55(0.38-0.71) |
| Rater A*rater 6 | 17 | 11 | 11 | 633 | 0.61 | 0.98 | 0.59(0.42-0.74) |
| Rater A*rater 7 | 20 | 8 | 8 | 636 | 0.71 | 0.99 | 0.70(0.54-0.83) |
| Rater A*rater 8 | 15 | 13 | 13 | 631 | 0.54 | 0.98 | 0.52(0.33-0.67) |
| Rater A*rater 9 | 10 | 18 | 18 | 626 | 0.36 | 0.97 | 0.33(0.15-0.48) |
| Rater A*rater 10 | 13 | 15 | 15 | 629 | 0.46 | 0.98 | 0.44(0.26-0.60) |
| Rater A*rater 11 | 19 | 9 | 9 | 635 | 0.68 | 0.99 | 0.67(0.5-0.80) |
| Rater A*rater 12 | 18 | 10 | 10 | 634 | 0.64 | 0.98 | 0.63(0.46-0.76) |
| Rater A*rater 13 | 17 | 11 | 11 | 633 | 0.61 | 0.98 | 0.59(0.42-0.74) |
| Rater A*rater 14 | 17 | 11 | 11 | 633 | 0.61 | 0.98 | 0.59(0.42-0.74) |
| Median (IQR) |  |  |  | 0.98 | 0.63 | 0.98 | 0.61(0.54-0.67) |
| **Set 2** |  |  |  |  |  |  |  |
| Rater A*rater 1 | 19 | 10 | 10 | 657 | 0.66 | 0.99 | 0.64(0.47-0.77) |
| Rater A*rater 2 | 22 | 7 | 7 | 660 | 0.76 | 0.99 | 0.75(0.61-0.87) |
| Rater A*rater 3 | 25 | 4 | 4 | 663 | 0.86 | 0.99 | 0.86(0.74-0.95) |
| Rater A*rater 4 | 22 | 7 | 7 | 660 | 0.76 | 0.99 | 0.75(0.61-0.86) |
| Rater A*rater 5 | 18 | 11 | 11 | 656 | 0.62 | 0.98 | 0.60(0.44-0.75) |
| Rater A*rater 6 | 22 | 7 | 7 | 660 | 0.76 | 0.99 | 0.75(0.61-0.86) |
| Rater A*rater 7 | 13 | 16 | 16 | 651 | 0.45 | 0.98 | 0.42(0.25-0.58) |
| Rater A*rater 8 | 21 | 8 | 8 | 659 | 0.72 | 0.99 | 0.71(0.55-0.84) |
| Rater A*rater 9 | 20 | 9 | 9 | 658 | 0.69 | 0.99 | 0.68(0.51-0.81) |
| Rater A*rater 10 | 15 | 14 | 14 | 653 | 0.52 | 0.98 | 0.50(0.32-0.64) |
| Rater A*rater 11 | 19 | 10 | 10 | 657 | 0.66 | 0.99 | 0.64(0.47-0.77) |
| Rater A*rater 12 | 21 | 8 | 8 | 659 | 0.72 | 0.99 | 0.71(0.55-0.84) |
| Rater A*rater 13 | 17 | 12 | 12 | 655 | 0.59 | 0.98 | 0.57(0.39-0.71) |
| Rater A*rater 14 | 13 | 16 | 16 | 651 | 0.45 | 0.98 | 0.42(0.25-0.58) |
| Median (IQR) |  |  |  |  | 0.68 | 0.99 | 0.66(0.55-0.75) |
| **Set 3** |  |  |  |  |  |  |  |
| Rater A*rater 1 | 24 | 5 | 5 | 662 | 0.83 | 0.99 | 0.82(0.70-0.93) |
| Rater A*rater 2 | 20 | 9 | 9 | 658 | 0.69 | 0.99 | 0.68(0.51-0.81) |
| Rater A*rater 3 | 25 | 4 | 4 | 663 | 0.86 | 0.99 | 0.86(0.74-0.95) |
| Rater A*rater 4 | 26 | 3 | 3 | 664 | 0.9 | 0.99 | 0.89(0.79-0.97) |
| Rater A*rater 5 | 25 | 4 | 4 | 663 | 0.86 | 0.99 | 0.86(0.74-0.95) |
| Rater A*rater 6 | 26 | 3 | 3 | 664 | 0.9 | 0.99 | 0.89(0.79-0.97) |
| Rater A*rater 7 | 22 | 7 | 7 | 660 | 0.76 | 0.99 | 0.75(0.61-0.87) |
| Rater A*rater 8 | 21 | 8 | 8 | 659 | 0.72 | 0.99 | 0.71(0.55-0.84) |
| Rater A*rater 9 | 22 | 7 | 7 | 660 | 0.76 | 0.99 | 0.75(0.61-0.87) |
| Rater A*rater 10 | 24 | 5 | 5 | 662 | 0.83 | 0.99 | 0.82(0.70-0.93) |
| Rater A*rater 11 | 23 | 6 | 6 | 661 | 0.79 | 0.99 | 0.78(0.64-0.89) |
| Rater A*rater 12 | 22 | 7 | 7 | 660 | 0.76 | 0.99 | 0.75(0.61-0.87) |
| Rater A*rater 13 | 26 | 3 | 3 | 664 | 0.9 | 0.99 | 0.89(0.79-0.97) |
| Rater A*rater 14 | 21 | 8 | 8 | 659 | 0.72 | 0.99 | 0.71(0.55-0.84) |
| Median (IQR) |  |  |  |  | 0.81 | 0.99 | 0.80(0.74-0.87) **∆∆, #** |

A, both raters scored criterion as being fulfilled; B, rater A scored criterion not fulfilled and rater 1 scored criterion as being fulfilled; C, rater A scored criterion as fulfilled and rater 1 scored criterion as not fulfilled; D, both raters agreed criterion not fulfilled; ppos, proportion of positive agreement; pneg, proportion of negative agreement; CI, confidence interval; IQR, interquartile range.

**∆∆** means p<0.001, Set 3 vs. Set 1; **#** means p<0.05, Set 3 vs. Set 2;
